# Supplementary figures and images for: Uptake of exogenous serine is important to maintain sphingolipid homeostasis in Saccharomyces cerevisiae
Source: PLoS Genet. 2020 Aug 26;16(8):e1008745. doi: 10.1371/journal.pgen.1008745 (PMC7478846; doi:10.1371/journal.pgen.1008745)

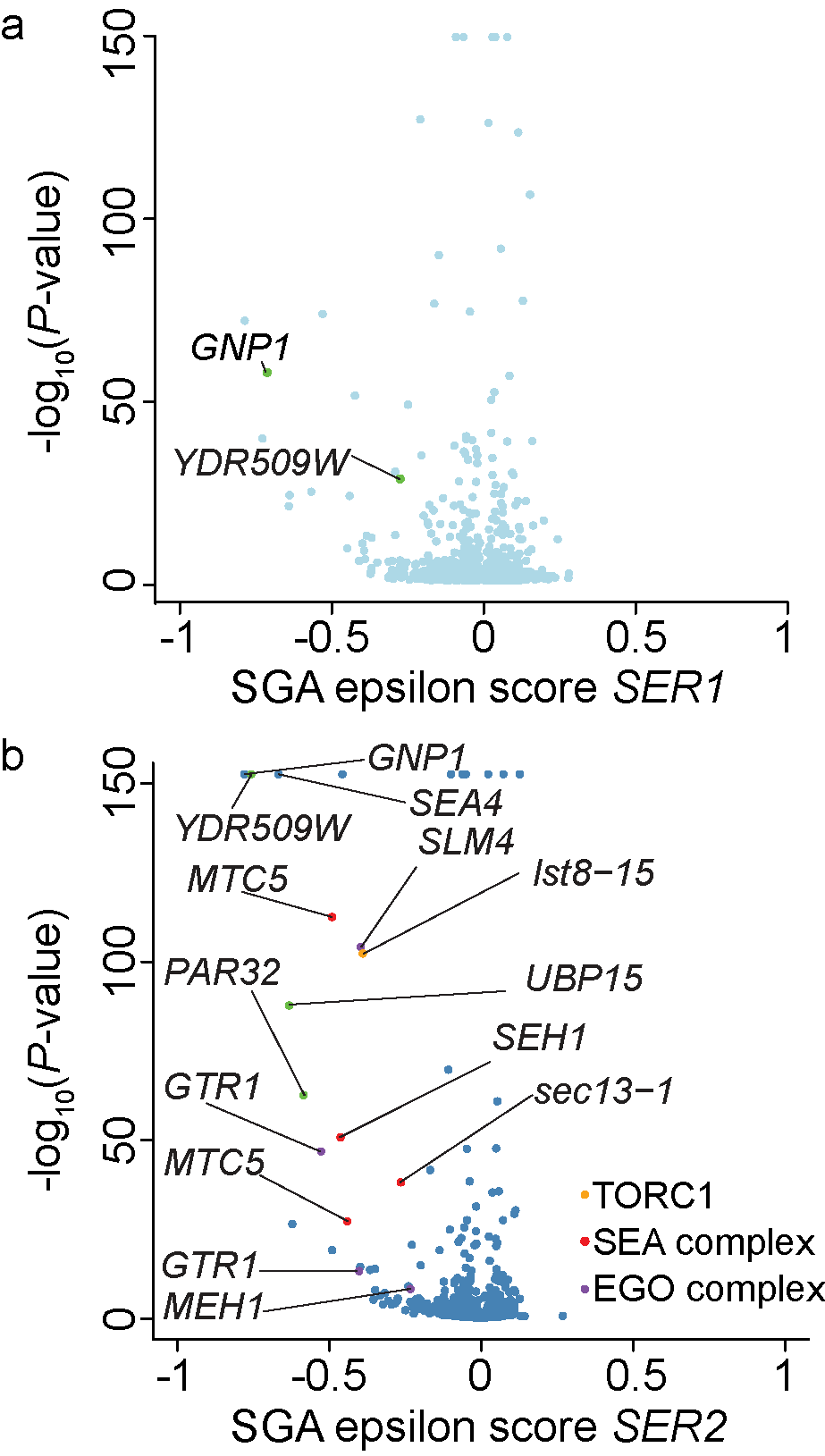

Supplement: S1 Fig — (a) The genetic interaction score (epsilon score) of SER1 is plotted against the negative LOG10 of the p-value of the interactions. The volcano plot shows significant negative genetic interactions on the left side of the plot. Data are taken from [24]. (b) The genetic interaction score (epsilon score) of SER2 is plotted against the negative LOG10 of the p-value of the interactions. The volcano plot shows significant negative genetic interactions on the left side of the plot. Dots are color coded according to the respective signaling pathways (orange–TORC1; read–SEA complex, purple–EGO complex, green–unknown signaling pathway). Data are taken from [24]. (TIF) [file pgen.1008745.s001.tif]

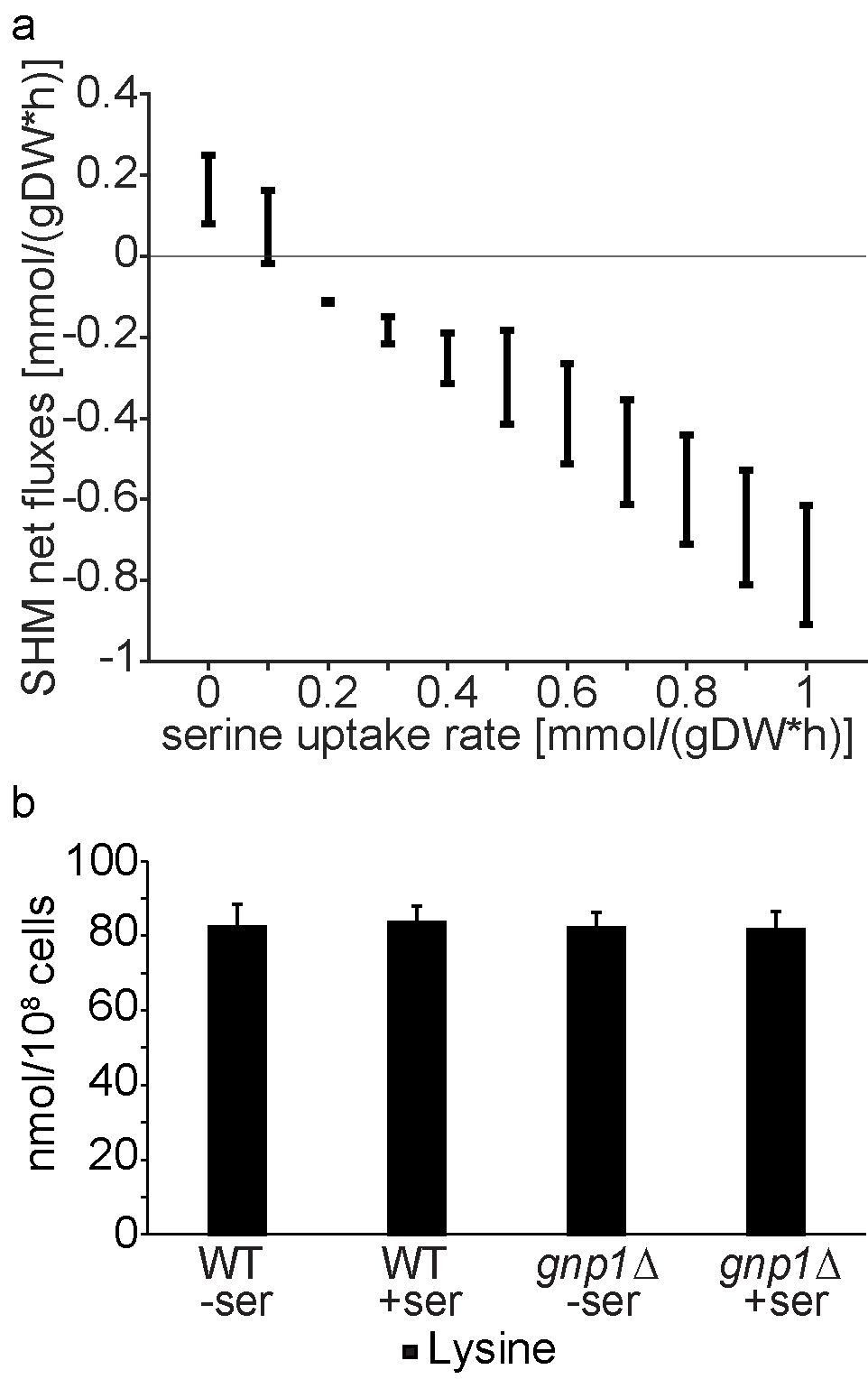

Supplement: S2 Fig — (a) Predicted serine hydroxymethyltransferases (Shm) net fluxes. Variability of net flux through Shm1 and Shm2 at varying serine uptake rates, as predicted by FVA. Positive and negative fluxes correspond to net production of serine and glycine, respectively. Fluxes and serine uptake rates are represented in mmol per gram dry weight per hour. (b) Cellular lysine concentrations. Prototroph WT and gnp1Δ cells were grown in synthetic media without amino acids and with and without serine. Lysine concentrations from whole cell lysates were analyzed by mass spectrometry. Error bars represent standard deviations. n = 3. (TIF) [file pgen.1008745.s002.tif]

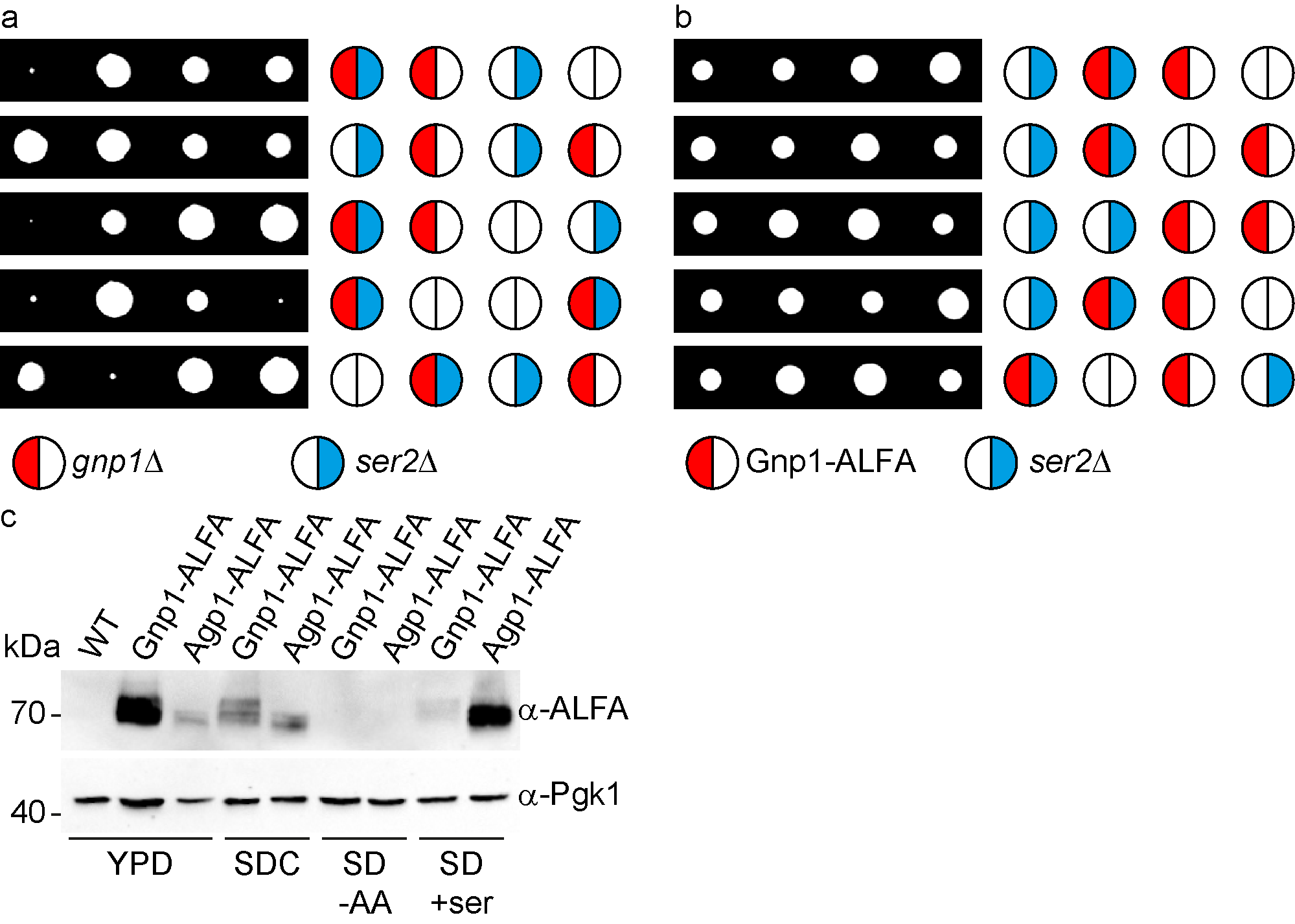

Supplement: S3 Fig — (a) Tetrad analysis of SEY6211 ser2Δ (blue) mutants crossed with SEY6210 gnp1Δ cells (red). (b) Tetrad analysis of SEY6211 ser2Δ (blue) mutants crossed with SEY6210 Gnp1-ALFA cells (red). (c) Expression level of Gnp1-ALFA and Agp1-ALFA. Cells were grown in YPD, SDC medium, SD medium without amino acids (AA) and SD media with serine. Equal amounts of cells were lysed and analyzed by western blotting using antibodies against the ALFA-tag or Pgk1 as a loading control. A wildtype strain was used as a control. (TIF) [file pgen.1008745.s003.tif]

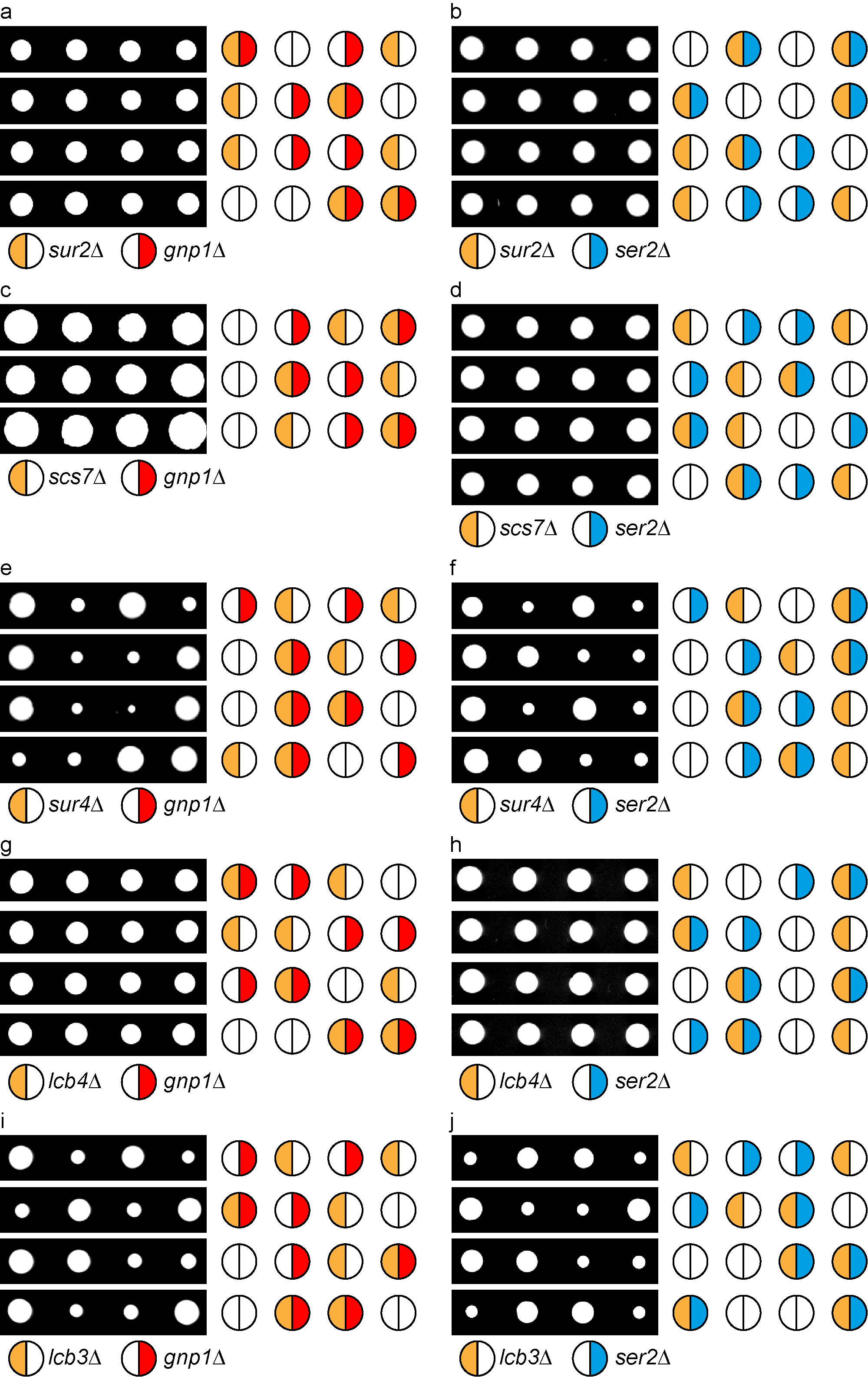

Supplement: S4 Fig — (a) Tetrad analysis of gnp1Δ (red) mutants crossed with sur2Δ (orange). (b) Tetrad analysis of ser2Δ (blue) mutants crossed with sur2Δ (orange). (c) Tetrad analysis of gnp1Δ (red) mutants crossed with scs7Δ (orange). d) Tetrad analysis of ser2Δ (blue) mutants crossed with scs7Δ (orange). (e) Tetrad analysis of gnp1Δ (red) mutants crossed with sur4Δ (orange). (f) Tetrad analysis of ser2Δ (blue) mutants crossed with sur4Δ (orange). (g) Tetrad analysis of gnp1Δ (red) mutants crossed with lcb4Δ (orange). (h) Tetrad analysis of ser2Δ (blue) mutants crossed with lcb4Δ (orange). (i) Tetrad analysis of gnp1Δ (red) mutants crossed with lcb3Δ (orange). (j) Tetrad analysis of ser2Δ (blue) mutants crossed with lcb3Δ (orange). (TIF) [file pgen.1008745.s004.tif]

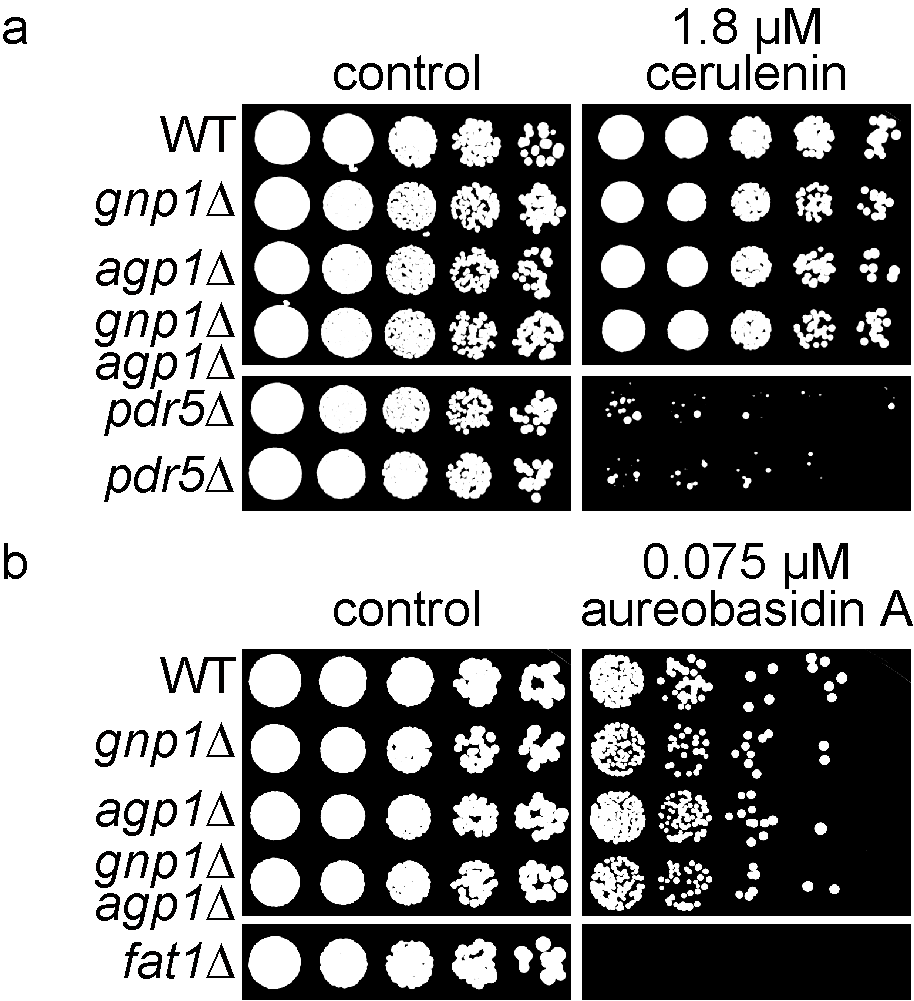

Supplement: S5 Fig — (a) Serial dilutions of WT, gnp1Δ cells, agp1Δ cells, gnp1Δ agp1Δ cells and two different clones of pdr5Δ cells on YPD plates. Control plates (left) and plates containing 1.8 μM cerulenin (right) were used. (b) Serial dilutions of WT, gnp1Δ cells, agp1Δ cells, gnp1Δ agp1Δ cells and fat1Δ cells on YPD plates. Control plates (left) and plates containing 0.075 μM Aureobasidin A (right) were used. (TIF) [file pgen.1008745.s005.tif]

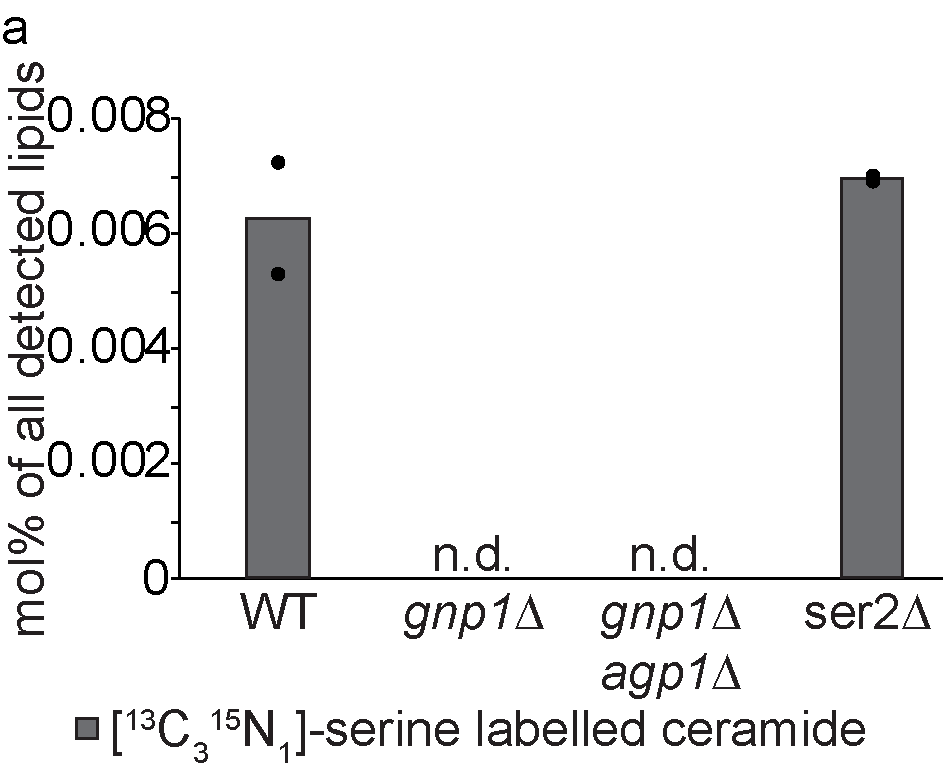

Supplement: S6 Fig — (a) Integration of [13C315N1]-serine into ceramides. Cells were labelled with [13C315N1]-serine and [2H6]-inositol over 90 minutes in YPD media. Lipids were extracted and analyzed via mass spectrometry. Displayed are the amounts of [13C315N1]-serine labelled ceramides of WT cells, gnp1Δ cells, gnp1Δagp1Δ cells and ser2Δ cells in mol% per all detected lipids. The average is displayed in bars. Dots correspond to the values of two independent experiments. (TIF) [file pgen.1008745.s006.tif]

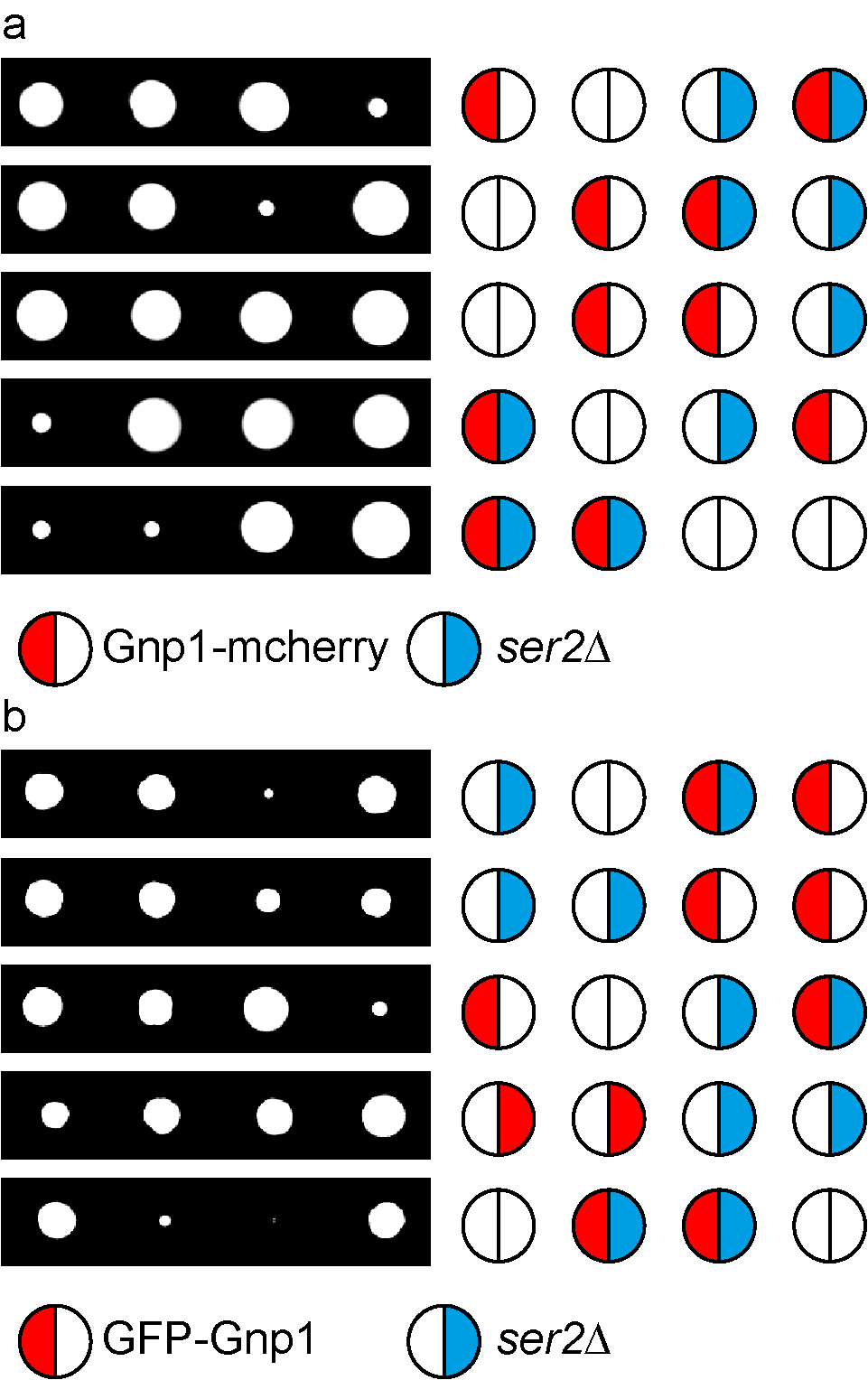

Supplement: S7 Fig — (a) Tetrad analysis of ser2Δ (blue) mutants crossed with Gnp1-mcherry (red). (b) Tetrad analysis of BY ser2Δ (blue) mutants crossed with BY GFP-Gnp1. (TIF) [file pgen.1008745.s007.tif]
